# The enigma of primary and secondary encapsulating peritoneal sclerosis

**DOI:** 10.1186/s12893-016-0198-2

**Published:** 2016-12-13

**Authors:** Hisham Allam, Omer Al Yahri, Sharon Mathew, Adham Darweesh, Ahmed Nafea Suliman, Sherif Abdelaziem, Mohamed Khairat, Adriana Toro, Isidoro Di Carlo

**Affiliations:** 1 Departments of General Surgery, Hamad General Hospital, Al Rayyan Road, 3050 Doha, Qatar; 2 Department of Radiology, Hamad General Hospital, Doha, Qatar; 3 Department of General Surgery, Barone I. Romeo Hospital, Patti, Messina Italy; 4 Department of Surgical Sciences and Advanced Technologies “G.F. Ingrassia”, University of Catania, Catania, Italy

**Keywords:** Encapsulating peritoneal sclerosis, Peritoneal encapsulation, Abdominal cocoon syndrome

## Abstract

**Background:**

Encapsulating Peritoneal Sclerosis (EPS) describes a variety of diseases that are frequently confused with different names and different etiopathogeneses. The aim of this article is to report personal experience of focusing on correct classification and the status of current diagnosis and treatment.

**Methods:**

A retrospective analysis was performed. Age, sex, ethnic origin, past medical history, symptoms and their duration, radiological tools and signs, laboratory tests, preoperative diagnosis, surgical approach, intraoperative findings, pathological findings, hospital stay, morbidity and mortality were studied.

**Results:**

A total of seven patients, including six males and one female, aged from 24 to 72 years were observed. Four patients had recurrent abdominal colic pain for 3 months, 1, 2 and 9 years; two patients also reported recurrent attacks but without any specification of the duration. All seven patients presented at the emergency department with abdominal pain that was mainly diffused over the entire abdomen. Six patients were submitted to a CT scan. Only in two patients was the diagnosis of EPS made preoperatively. All seven patients were submitted to open surgery. The hospital stay was between 4 and 60 days. One patient had morbidity, and one patient died of MOF.

**Conclusions:**

Currently, the correct identification of EPS is more easily possible than in the past, but the diagnosis is still a challenge. Surgery must be performed as soon as possible to avoid a poorer quality of life.

## Background

Encapsulating peritoneal sclerosis (EPS) is an uncommon chronic syndrome, usually presenting clinically as intermittent, acute or sub-acute gastrointestinal obstruction [1]. EPS can be divided into primary and secondary [2]. Primary EPS is also defined as idiopathic [3]. Primary EPS have been also defined in 1978 as abdominal cocoon syndrome [4]. The cause of primary EPS is still unknown; [4, 5].

Secondary EPS is related to many conditions and causes; the most common is peritoneal dialysis (Table 1).

**Table 1:** Classification of different aspects of peritoneal encapsulation

1) Peritoneal encapsulation
2) Sclerosing encpsulating peritonitis
a) Primary or idiopatic or abdominal cocoon syndrome
b) Secondary

Both forms (primary and secondary) are different from peritoneal encapsulation (PE). This disease was reported for the first time in 1868 by Cleland [6], and it is represented by an accessory peritoneal membrane. More than the result of inflammation should be considered with a duplication of the peritoneum; this is found incidentally in the majority of cases. The peritoneal membrane in this disease is secured laterally to the ascending and descending colon, cranially to the transverse mesocolon, and caudally to the posterior parietal peritoneum. It has two openings: one at the entrance of the intestine at the duodenojejunal junction, and the other at the ileocecal junction for the exit of the last loop. The absence of sclerosis represents the difference with the EPS, in fact the coverage of the intestine is only a layer of serosa membrane like the peritoneum.

Many tools can be used to diagnose EPS. Instead of utilizing the past medical history and the radiological finding, preoperative diagnosis remains challenging, and in a high percentage of cases, the diagnosis is reached at the time of the surgical procedure.

The aim of this article is to report a valuable number of patients who were observed in our hospital, focusing on correct classification and the status of current diagnosis and treatment.

## Methods

A retrospective analysis was performed on patients admitted to the Hamad General Hospital Department of Surgery, General Surgery section, in the last 10 years from January 2005 to September 2015. Age, sex, ethnic origin, past medical history, symptoms and their duration, radiological tools and signs, laboratory tests, preoperative diagnosis, surgical approach, intraoperative findings, pathological examinations, hospital stay, morbidity and mortality were studied. After this primary evaluation, all patients were classified by primary or secondary EPS, and all of the data have been revaluated to note the differences.

## Results

**Fig. 1:**
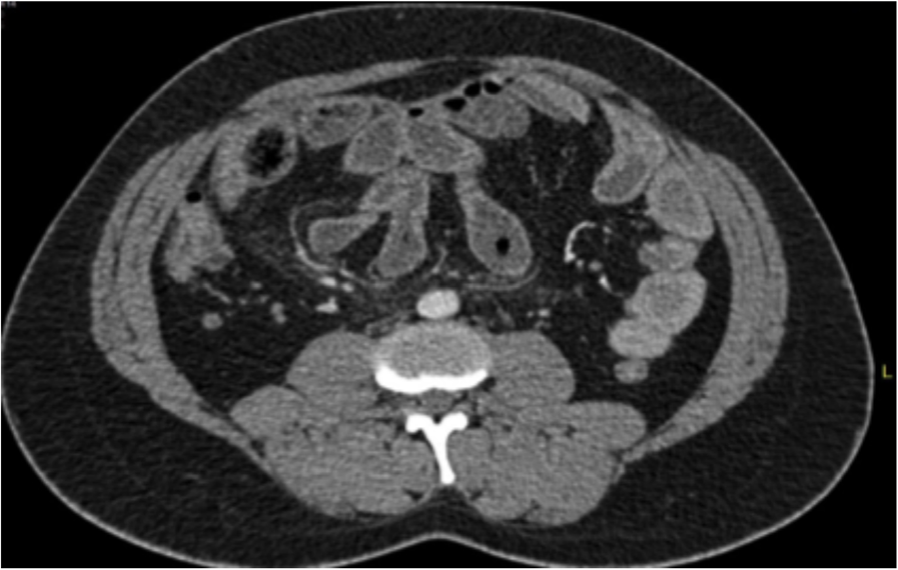
Contrast-enhanced CT scan of the abdomen showing a conglomerate of multiple small-bowel loops seen in the center of the abdomen, surrounded by a thick enhanced saclike structure

**Fig. 2:**
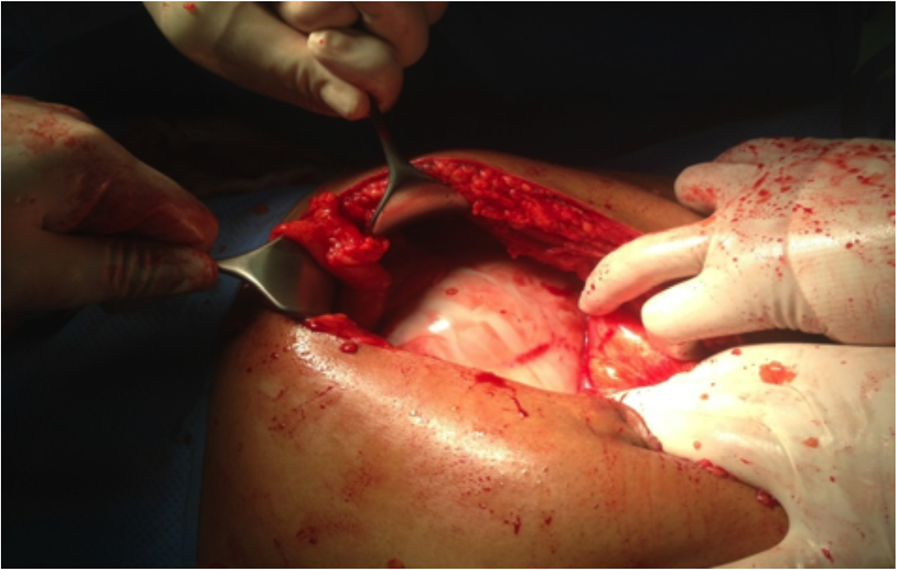
View when entering the abdominal cavity

**Fig. 3:**
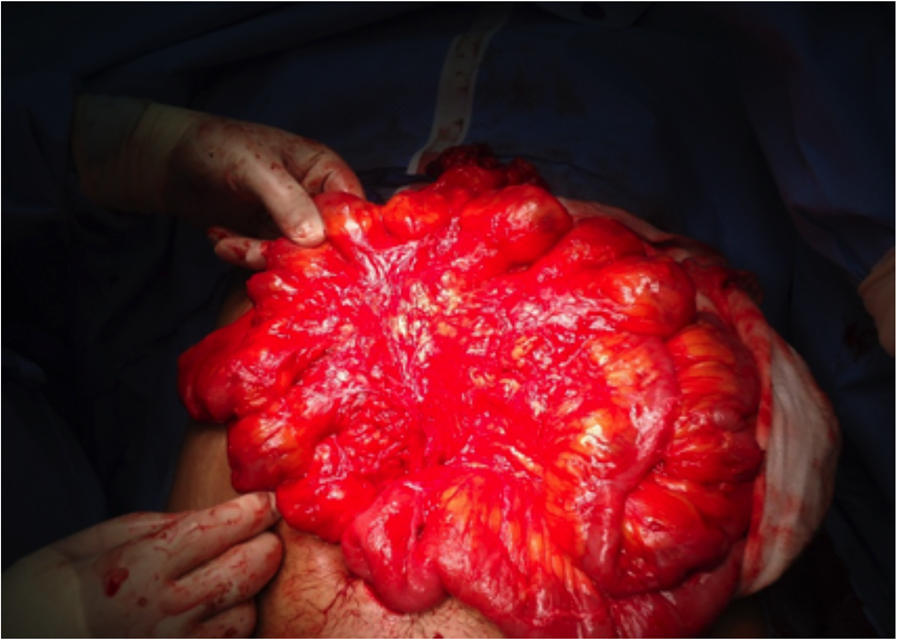
After division of the membrane and freeing the bowel loops

In the period of the study, 10 patients were observed. Three of these patients were previously published as case reports [6, 7], and they will not be further reported on here. Thus, we have analyzed the remaining seven patients who have not yet been reported. The patients were six males and one female, aged from 24 to 72 years (mean age 44.5 years). The nationalities of the patients were three Egyptians, two Indians, one Sudanese and one Nepalese. Three patients had no comorbidities and were considered to have primary EPS; the remaining four patients had comorbidities. In one case, the patient had a Mediterranean fever that was diagnosed more than 10 years ago. In three cases, the patients were submitted to peritoneal dialysis, in one case for 32 months and in the second case, for 8 months; the duration was not reported in the last case. All seven patients presented at the emergency department with abdominal pain that was mainly diffused over the entire abdomen; in addition to the main symptoms, five patients had nausea, vomiting and constipation. One patient had only diffuse abdominal pain and anorexia. Four patients had recurrent abdominal colic pain for 3 months, 1, 2 and 9 years; two patients also reported recurrent attacks but without any specification of the duration. One patient had no history of a previous attack of abdominal pain. Six patients were submitted to a CT scan. Only in two patients was the diagnosis made preoperatively: in one case, as a partial encapsulation and in the second one, as a complete encapsulation of the small bowel (Fig. 1). In the remaining four cases, the diagnosis was of intestinal occlusion without any specifications. The last patient was submitted to an X-ray examination that showed intestinal occlusion but the diagnosis was erroneous because the patient presented a bulk on the right side of the umbilicus that was diagnosed as an obstructed Spigelian hernia. Five patients presented with a slight elevation of WBC that was normal in one patient and not reported in the last one. All seven patients were submitted to open surgery. Five patients had the entire small bowel encapsulated (Fig. 2); one patient had an encasement of the last part of the jejunum, ileum, appendix and cecum. The last patient had an encasement of the small bowel and cecum with multiple perforations between the last ileal loop and the cecum. All patients were submitted to excision of the membranes and adhesiolysis of the loops (Fig. 3), except the patient who was affected by multiple perforations that was submitted to excision of the membrane, adhesiolysis and right hemicolectomy. In all seven cases, pathological examinations showed the presence of hyalinized membranous fibrocollagenous tissue compatible with a diagnosis of EPS. The hospital stay was between 4 and 60 days. One patient had morbidity; the patient came back to the hospital after 1 week with the symptoms of intestinal occlusion. The patient was treated with bowel rest and parenteral nutrition, and he resolved spontaneously after 4 days. Then, he was discharged and no further complaints have been recorded. One patient died after the initial resection of the last loops of the ileum and the right colectomy. The patient developed a wound infection, wound dehiscence and purulent peritonitis with septic shock. He underwent a second operation 2 weeks after the first procedure, and a couple of small bowel perforations were found. Both the first and second procedures were very difficult because of the extensive adhesions between the bowel loops. Instead of several operations and multiple treatments, the patient developed an entero-cutaneous fistula and consequently abdominal sepsis and septic shock, and he died as a result of multiple organ failure after 60 days of hospitalization.

All of the analyzed data have been used after classifying the patients as having primary and secondary EPS. All of the differences in the two groups are shown in Table 2.

**Table 2:** Mean characteristics of both group of the patients: primary and secondary SEP

	Primary SEP	Secondary SEP
Nb Pts	3	4
Sex	3 males	3 males, 1 female
Age	33 years mean age (30–36)	53,5 mean age (24–72)
Nationality	1 Indian	2 Egiptian
	1 Egyptian	1 Sudani
	1 Nepali	1Indian
Comorbidities	No comorbidities	3pts CKD with PD
		1 pt Mediterranean fever
Symptoms of presentation at emergency	2pts abdominal pain, nausea, vomiting, constipation	3pts abdominal pain, nausea, vomiting, constipation
	1 pt abdominal pain	1 pt abdominal pain and anorexia
Duration of symptoms	1^st^ 2 years	1^st^ 9 years
	2^nd^ 1 year	2^nd^ 3 months
	3^th^ no previous attack	2 pts previous attack but not specified the time
Methods of diagnosis	1 pt X-ray	4 pts CT scan
	2 pts CT scan	
Accuracy of diagnosis	2 pts SEP	4 pts general intestinal occlusion
	1 pt Spigelian hernia	
Preoperative WBC	2 pts increased slightly	3 pts increased slightly
	1 pt not reported	1 pt normal
Presentation at surgery	1 pt encasement of entire small bowel, cecum, appendix	3pts encasement of entire small bowel
	2 pts encasement of entire small bowel	1 pt encasement of entire small bowel, an cecum
Surgical procedure	3 pts excision of membranes and adhesiolisis	3 pts exicision of membranes and adhesiolisis
		1 pt excision of membranes, adhesiolisis and RH
Hospitalization	1^st^ 10 days	1^st^ 60 days
	2^nd^ 4 days	2^nd^ 7 days
	3^th^ 4 days	3^th^ and 4^th^ 4 days
Morbidity	1 pt Early post operative small bowel obstruction	None
Mortality	None	1 pt MOF

*Nb* number, *Pts* patients, *CKD* Chronic Kidney Disease, *PD* peritoneal dialysis, *WBC* White Blood Cells, *CT* Computed Tomography, *MOF* Multi Organ Failure

## Discussion

One of main issues of peritoneal encapsulation and EPS is the different forms and different definitions that are frequently used in the wrong manner, resulting in confusion regarding all of the forms. Differentiation is simply done by pathological examinations of the membranes. In the case of PE, the membrane is covered by mesothelium because it is an accessory peritoneal membrane that is a kind of malformation. In contrast, in primary and secondary EPS, the membrane that encases the intestine is fibrous, containing inflammatory cells; this is a consequence of the fact that this condition is acquired as a result of an inflammation of the peritoneal serosa that can be stimulated by many factors. EPS can be secondary to the activity of multiple factors that can be responsible for the inflammation of a systemic activity [8–11] or for local and/or systemic irritant factors [12–14]. Dialisys represents the most common cause of secondary EPS and it is well established that is related to the chronic exposure to bioincompatible dialysate. This exposure may represent the initial step for the fibrotic process, that may evolve until EPS [15]. In fact multiple and repetitive episodes of tissue injury with consequent inflammation and deposit of fibrin, permit to alert the mesothelial cells and fibroblast, with development and deposition of matrix that represents the precursor of EPS [15].

EPS can also be primary or idiopathic and can also be defined as “abdominal cocoon syndrome”; in this case, the cause is still unknown and it can present both in adolescent and in adults. Different from secondary EPS, this form can be without adhesions between the loops [16]. The abdominal cocoon has differentiation in relation to the extent of the encapsulation and the affected organ (Table 3) [17, 18]. Some case reports have confused primary abdominal cocoon syndrome and peritoneal encapsulation in the past [19–22]. The etiology of primary EPS is still debated. Both sexes are at risk, but, as in the major report in the literature [23], our experience shows that males are the predominant sex. Primary EPS has been reported in adolescent females from tropical or subtropical countries but also adult cases have been reported from temperate zone as well [23]. One of the main hypotheses circulating about the etiology has been retrograde menstruation with consequent viral infection [23]. This hypothesis has not still been validated as a result of the fact that men and premenopausal women are also affected by this form of the disease.

**Table 3:** Classification and definition of different type of abdominal cocoon

Type 1	Partial encasement of intestine by a fibrocollagenous membrane
Type 2	Complete encasement of intestine by a fibrocollagenous membrane
Type 3	Complete encasement of the whole intestine and other orgen like appendix, ascending colon cecum, ovaries by a fibrocollagenous membrane.

In our case series, all three cases of primary EPS were male patients. All are young and this reflects the fact that the country of Qatar is full of young male expatriates that are construction laborers; none of the three patients came from tropical or subtropical countries. Additionally, in the second group, not all patients were from tropical or subtropical countries, but all of these patients have a comorbidity that can be considered as the cause of the secondary EPS. In this second group, in contrast, the age reflects the comorbidities that are the cause of the EPS except in the patient who was affected by the Mediterranean familial fever (a recurrent polyserositis mainly affecting the peritoneum) who was aged 24 years; the other three patients were aged more than 50 years. Obviously, our case series is limited in number, but the fact that all patients do not come from tropical or subtropical countries leaves open the possibility regarding the origin of the countries.

The phisiopatology of EPS is based on alteration of the intestinal functions associated to the reabsorptive functions. This fibrotic process may cause a reduction in motility, up to ileus, encapsulation and the destruction of the myenteric plexus [24].

The preeminent clinical signs of this disease are abdominal pain, nausea, vomiting and occasional constipation. Recurrent episodes of peritonitis, with negative culture, spontaneously regressed, in association with anemia, low albumin and high C-reactive protein, can be found in case of EPS [15]. These are non-specific signs of acute abdominal obstruction, but clinically can help the past medical history. In fact, all of these patients, as in our case series, had previous attacks of colic pain, demonstrating that the majority of patients have a repetitive chronic symptomatology. This can happen for many years before the right diagnosis is achieved. In patients with primary EPS, the positive anamnesis for several colic pain occurrences in the past is the only sign that can help. In contrast, in secondary EPS, the previous attack of colic pain in association with the risk factors can be more useful in formulating the clinical diagnosis.

In effect, because the disease is very rare, the correct diagnosis with radiological tools depends both on the armamentarium and the skillfulness of the radiologist. The most important signs are the thickening of the peritoneum, bowel encapsulation, intestinal obstruction, presence of cocoon, and detection of calcification. X-Ray examination may show air fluid levels and bowel obstruction in association with abdominal calcifications, but all these signs are not sufficient to diagnose EPS [25]. A CT scan is the most useful in establishing a preoperative diagnosis. The appearance of a conglomeration of all small bowel loops encased by a capsule dense with contrast free in the periphery can be considered characteristics of this disease using a CT scan (Fig. 1) The contrast-enhanced CT may have a sensitivity of 100% and a specificity of 94% for diagnosing EPS [26]. Bowel loops collected into the centre of the abdomen as a sign of adhesions, enhanced thickened peritoneum with calcifications [27] and lobulated ascites in the omentum or between the bowel loops are the signs shown by Magnetic Resonance Imaging [28]. Surprisingly, in our case series the correct diagnosis was made in only two cases of primary EPS. In contrast, all the patients of the secondary EPS, (that had more clinical helping factors) had only a generic intestinal occlusion diagnosis. An MRI was reported only one time, and the result was considered equal to the CT scan [29]. Ultrasonography cannot help in the specific diagnosis; they can only permit a generic diagnosis of abdominal occlusion [2].

The histopathology of this disease is not pathognomonic, and a diagnosis after the resected specimen is still achieved in concomitance to the clinical and radiological findings.

There is no consensus about the treatment of EPS.

Patients with mild abdominal pain could be treated conservatively as long as the symptoms regress with nasogastric tube decompression, bowel rest and nutritional support [6]. These patients usually have a malnutrition status that reflects the chronicity of the disease. Total parenteral nutrition does not have any curative effect and it have to be used in patient to restore the nutritional status especially before surgery [30]. Additionally, continuous attacks of abdominal pain can result in patients’ malnutrition because of a limitation of food intake as a result of nausea and vomiting. This also reflects a poor quality of life, especially as in our young patient, who was affected by familial Mediterranean fever in which the colicky attacks are present for 9 years. A special case of secondary EPS can be treated with steroids, tamoxifen or sirolimus [31]. Surgical treatment has improved in the last decades. Peritonectomy and careful lysis with resection of the peritoneum and fibrous tissue, in association with separation of adhesions to release the bowel, represents the treatment of choice. Post operative mortality ranges from 19 to 34.5%, [32] with recurrence rate at around 25%. Some Authors have used the Noble plicature with a decrease in the recurrence rate to 12,3% [33].

An immediate early postoperative small bowel obstruction can be recorded in the immediate post-operative period because a long operative dissection of the loops frequently results in edema. This very early post-operative complication represents a very difficult reoperation for the patient [34] and should be treated by bowel rest and parenteral nutrition. The use of somatostatin, in association with a low dose of steroid, has been suggested for treatment of this early complication [23]. Additionally, intraoperative stenting of the intestinal loops has been suggested to improve post-operative results and avoid the previously listed complications [23].

In our experience patients have been operated as soon as possible. In fact, the disease does not regress and the quality of life of patients can be poor in relation to the intensity and frequency of the attacks. Furthermore, patients having attacks for many years who have not submitted to surgery can have a more difficult adhesive situation that increases the morbidity and mortality as reported in our patients. An incidental appendectomy is suggested by some authors to avoid a difficult appendectomy if the patient developed acute appendicitis [35] later on. All our patients were treated surgically with the excision of all membranes (Fig. 2) and adesiolysis (Fig. 3).

## Conclusions

Currently, the correct identification of the form of EPS and PE is more easily possible than in the past, but the diagnosis is still a challenge. In our opinion, surgery has to be done as soon as possible to avoid a poor quality of life with recurrent episodes of occlusion and difficult adhesions that can cause morbidity and mortality.

## Abbreviations

CT Scan: Computerized tomography scan
EPS: Encapsulating peritoneal sclerosis
MRI: Magnetic resonance imaging
PE: Peritoneal encapsulation
WBC: White blood cell

## References

[1] Augustine T, Brown PW, Davies SD, Summers AM, Wilkie ME (2009). Encapsulating peritoneal sclerosis: clinical significance and implications. Nephron Clin Pract.

[2] Akbulut S, Yagmur Y, Babur M (2014). Coexistence of abdominal cocoon, intestinal perforation and incarcerated Meckel’s diverticulum in an inguinal hernia: a troublesome condition. World J Gastrointest Surg.

[3] Akbulut S (2015). Accurate definition and management of idiopathic sclerosing encapsulating peritonitis. World J Gastroenterol.

[4] Foo KT, Ng KC, Rauff A, Foong WC, Sinniah R (1978). Unusual small intestinal obstruction in adolescent girls: the abdominal cocoon. Br J Surg.

[5] Browne LP, Patel J, Guillerman RP, Hanson IC, Cass DL (2012). Abdominal cocoon: a unique presentation in an immunodeficient infant. Pediatr Radiol.

[6] Al-Thani H, El Mabrok J, Al Shaibani N, El-Menyar A (2013). Abdominal cocoon and adhesiolysis: a case report and a literature review. Case Rep Gastrointest Med.

[7] Bassiouny IE, Abbas TO (2011). Small bowel cocoon: a distinct disease with a new developmental etiology. Case Rep Surg.

[8] Basu A, Sukumar R, Sistla SC, Jagdish S (2007). “Idiopathic” abdominal cocoon. Surgery.

[9] Rokade ML, Ruparel M, Agrawal JB (2007). Abdominal cocoon. J Clin Ultrasound.

[10] Tan FL, Loh D, Prabhakaran K (2005). Sclerosing encapsulating peritonitis in a child secondary to peritoneal dialysis. J Pediatr Surg.

[11] Vijayaraghavan SB, Palanivelu C, Sendhilkumar K, Parthasarathi R (2003). Abdominal cocoon: sonographic features. J Ultrasound Med.

[12] Maguire D, Srinivasan P, O’Grady J, Rela M, Heaton ND (2001). Sclerosing encapsulating peritonitis after orthotopic liver transplantation. Am J Surg.

[13] Sigaroudinia MO, Baillie C, Ahmed S, Mallucci C (2008). Sclerosing encapsulating peritonitis—a rare complication of ventriculoperitoneal shunts. J Pediatr Surg.

[14] Kaushik R, Punia RP, Mohan H, Attri AK (2006). Tuberculous abdominal cocoon—a report of 6 cases and review of the Literature. World J Emerg Surg.

[15] Moinuddin Z, Summers A, Van Dellen D, Augustine T, Herrick SE (2015). Encapsulating peritoneal sclerosis-a rare but devastating peritoneal disease. Front Physiol.

[16] Cleffken B, Sie G, Riedl R, Heineman E (2008). Idiopathic sclerosing encapsulating peritonitis in a young female-diagnosis of abdominal cocoon. J Pediatr Surg.

[17] Tannoury JN, Abboud BN (2012). Idiopathic sclerosing encapsulating peritonitis: abdominal cocoon. World J Gastroenterol.

[18] Wei B, Wei HB, Guo WP, Zheng ZH, Huang Y, Hu BG, Huang JL (2009). Diagnosis and treatment of abdominal cocoon: a report of 24 cases. Am J Surg.

[19] Sayfan J, Adam YG, Reif R (1979). Peritoneal encapsulation in childhood. Case report, embryologic analysis, and review of literature. Am J Surg.

[20] Sieck JO, Cowgill R, Larkworthy W (1983). Peritoneal encapsulation and abdominal cocoon. Case reports and a review of the literature. Gastroenterology.

[21] Devay AO, Gomceli I, Korukluoglu B, Kusdemir A (2006). An unusual and difficult diagnosis of intestinal obstruction: the abdominal cocoon. Case report and review of the literature. World J Emerg Surg.

[22] Devay AO, Gomceli I, Korukluoglu B, Kusdemir A (2008). An unusual and difficult diagnosis of intestinal obstruction: the abdominal cocoon. Case report and review of the literature. World J Emerg Surg.

[23] Li N, Zhu W, Li Y, Gong J, Gu L, Li M, Cao L, Li J (2014). Surgical treatment and perioperative management of idiopathic abdominal cocoon: single-center review of 65 cases. World J Surg.

[24] Kawanishi H, Kawaguchi Y, Fukui H, Hara S, Imada A, Kubo H, Kin M, Nakamoto M, Ohira S, Shoji T (2004). Encapsulating peritoneal sclerosis in Japan: a prospective, controlled, multicenter study. Am J Kidney Dis.

[25] Choi JH, Kim JH, Kim JJ, Jin SY, Choi DL (2004). Large bowel obstruction caused by sclerosing peritonitis: contrast-enhanced CT findings. Br J Radiol.

[26] Vlijm A, van Schuppen J, Lamers AB, Struijk DG, Krediet RT (2011). Imaging in encapsulating peritoneal sclerosis. NDT Plus.

[27] Hüser N, Stangl M, Lutz J, Fend F, Kreymann B, Gaa J (2006). Sclerosing encapsulating peritonitis: MRI diagnosis. Eur Radiol.

[28] Lien YC, Kuo CC, Liu KL, Hung KY, Huang TM, Huang JW (2009). Clinical images: encapsulating peritoneal sclerosis. CMAJ.

[29] Jovani M, Baticci F, Bonifacio C, Omodei PD, Malesci A (2014). Abdominal cocoon or idiopathic encapsulating peritoneal sclerosis: magnetic resonance imaging. Dig Liver Dis.

[30] Campbell R, Augustine T, Hurst H, Pararajasingam R, van Dellen D, Armstrong S, Bartley C, Birtles L, Summers A (2015). Anthropometrics identify wasting in patients undergoing surgery for encapsulating peritoneal sclerosis. Perit Dial Int.

[31] Moustafellos P, Hadjianastassiou V, Roy D, Velzeboer NE, Maniakyn N, Vaidya A, Friend PJ (2006). Tamoxifen therapy in encapsulating sclerosing peritonitis in patients after kidney transplantation. Transplant Proc.

[32] Ulmer C, Braun N, Rieber F, Latus J, Hirschburger S, Emmel J, Alscher MD, Steurer W, Thon KP (2013). Efficacy and morbidity of surgical therapy in late-stage encapsulating peritoneal sclerosis. Surgery.

[33] Kawanishi H (2012). Surgical and medical treatments of encapsulation peritoneal sclerosis. Contrib Nephrol.

[34] Sajja SB, Schein M (2004). Early postoperative small bowel obstruction. Br J Surg.

[35] Kayastha K, Mirza B (2012). Abdominal cocoon simulating acute appendicitis. APSP J Case Rep.

